# Quantitative trait loci and genome-wide association study identifies novel loci for plant height in pepper (*Capsicum* spp.)

**DOI:** 10.3389/fpls.2026.1934965

**Published:** 2026-10-01

**Authors:** Geon Woo Kim, Abhinandan S. Patil, Kyeong-Seok Lee, Min-Young Kang, Hea-Young Lee, Solomon Abate Mekonnen, Muhammad Irfan Siddique, Koeun Han, Jelli Venkatesh, Jin-Kyung Kwon, Byoung-Cheorl Kang

**Affiliations:** Department of Agriculture, Forestry and Bioresources, Research Institute of Agriculture and Life Sciences, Plant Genomics and Breeding Institute, College of Agriculture and Life Sciences, Seoul National University, Seoul, Republic of Korea

**Keywords:** *Capsicum*, genome-wide association study, linkage disequilibrium, main stem, plant height, quantitative trait locus

## Abstract

Plant height (PH) is a key trait that influences photosynthetic efficiency, early maturity, lodging resistance, and crop yield. The development of plant factories and mechanical harvesting has increased the demand for peppers (*Capsicum* spp.) with suitable plant architecture. In this study, we performed quantitative trait locus (QTL) analysis and genome-wide association studies (GWAS) to determine the genetic basis of PH in pepper. For QTL analyses, 117 F_2_ individuals derived by crossing *C. annuum* ‘Jeju’ and ‘Micropep’ were used for PH analysis. Another population of 154 F_2_ plants derived from crossing *C. annuum* ‘Takanotsume’ and ‘Micropep’ was used to analyze main stem length (MSL), number of main stem nodes (NMSN), and average node length of the main stem (ANLMS). Of these, 153 individuals were used for genetic map construction and QTL mapping. After genotyping-by-sequencing and filtering, 1,066 SNPs from the JM population and 1,446 from the TM population were retained for bin-map construction. QTL mapping was conducted with 451 bin markers for JM and 518 for TM. The GWAS used 25,126 SNPs on chromosomes 1–12 in the core collection. Composite interval mapping revealed QTLs for PH on chromosomes 1 and 11 in the JM population. QTL analysis in the TM population identified a significant NMSN QTL on chromosome 3 and a significant MSL QTL on chromosome 1. GWAS additionally identified significant SNPs for PH, NMSN, ANLMS, and MSL in the core collection. Genes near these SNPs were predicted, based on sequence similarity to characterized genes in other species, to include transcription factors, cell-wall-remodeling proteins, metabolic enzymes, and developmental regulators. Neither of the significant TM QTLs overlapped an LD block containing significant GWAS SNPs for the corresponding trait. These findings provide linkage regions, association signals, and predicted candidate genes for plant height and main stem traits in pepper, which will require validation in independent populations before they can be used in breeding.

## Introduction

Pepper is an important vegetable and spice crop rich in various compounds such as capsaicinoids, carotenoids, volatile oils, proteins, minerals, and vitamins (Krishna De, 2003; Bosland and Votava, 2012). In an evolutionary context, three independent cultivated *Capsicum* species, *Capsicum annuum*, *C. chinense*, and *C. frutescens* which hold a selective advantage of better canopy development, resource acquisition, competition, and phenotypic plasticity have emerged from wild (sub)shrubby-herbaceous *Capsicum* complex (Moscone et al., 2007; Ford, 2014). Among morphometric traits, plant height (PH) is an important component of plant architecture, and it influences light interception and utilization, photosynthetic efficiency, early maturity, lodging resistance, and yield improvement of pepper (Pickersgill, 2007). In recent years, pepper breeders put greater emphasis on the improvement of compact plant architecture for high-density planting and lodging resistance in field and greenhouse conditions (Choi et al., 2010). By contrast, mechanical harvesting requires a different combination of characteristics, including sufficient plant height, a high first bifurcation, a large stem diameter, an erect growth habit, few lateral shoots, a firm root system, and a narrow branching angle (Wall et al., 2003; Funk et al., 2011). These two objectives are therefore not equivalent, and a single ideotype does not satisfy both. What they share is a dependence on how the main stem is built, that is, on its total length, the number of nodes it carries, and the length of each node. The present study measured these components separately rather than plant height alone, so that the resulting loci can be interpreted for either objective.

To date, only a few genetic studies have reported QTLs for plant architecture traits with the use of low-density markers (Chaim et al., 2001b; Dwivedi et al., 2015) and only a few studies (Han et al., 2016) used high-resolution markers derived from next-generation sequencing. Using restriction fragment length polymorphism (RFLP), amplified fragment length polymorphism (AFLP), and random amplified polymorphism DNA (RAPD) markers and an F_3_ population derived from a cross between two *C. annuum* genotypes, the bell-type cultivar ‘Maor’ and the Indian small-fruited line ‘Perennial’. Chaim et al. (2001b) reported the QTLs for PH on linkage groups (LGs) 2, 3, 4, 6, and 8, of which QTLs on LGs 3, 4, and 6 were consistent. In another study, Dwivedi et al. (2015) identified a single major effect of QTL for PH on LG 5 using SSR, SCAR, and RAPD markers in the F_8:9_ RIL population derived from a cross between the *C. annuum* ‘California Wonder’ and the ‘LCA235’. Using high-density SNP markers, Han et al. (2016) reported several PH-related QTLs on chromosomes 2, 4, 6, 7, and 8 and two major QTLs for lateral branch number on chromosome 2 in the F_7:10_ RIL population developed from a cross between the ‘Perineal’ and the ‘Dempsey’. QTLs for plant growth traits in pepper were also identified in an intraspecific *C. annuum* doubled haploid population for several plant architectural traits (Mimura et al., 2010). However, none of these QTLs for plant architecture have been compared with association signals from a genetically diverse panel of pepper.

Based on the functional studies of known candidate genes or homologs from other species in the Solanaceae family, the role of several genes, such as the *Capsicum SP-like* gene (*CaSP*), has been proven to control determinate growth (Kim et al., 2006). Similarly, the use of many induced and natural mutations in pepper enabled the allele mining of key genes controlling sympodial (determinate) shoot development (*FASCICULATE* and *CaJOINTLESS*), flower promoting (*Ca-VIL1*, *CaS*, *Ca-LEAFY*, and *Ca‐ANANTHA*), flower repressing (*CaAP2*), and axillary branching (*CaBLIND* and *CaHAM*) (Lippman et al., 2008; Elitzur et al., 2009; Jeifetz et al., 2011; Cohen et al., 2012; David-Schwartz et al., 2013; Borovsky et al., 2015). Meristem identity genes not only control and determine the fate of floral organs but also partly regulate plant shoot architectural organs through activating homeotic genes (Jack, 2004; Krizek and Fletcher, 2005). In previous studies, QTLs related to traits such as lateral branch number, plant width, and internode length were identified (Yarnes et al., 2013; Han et al., 2016). Furthermore, several QTLs related to stem length, internode length, axis growth speed, and internode growth time were identified in chromosomes 2, 4, 9, and LG47 in a recombinant inbred line (RIL) population derived from a cross between the ‘Yolo Wonder’ and the ‘CM334’ (Barchi et al., 2009). However, further studies are needed to understand their genetic basis and reveal novel genetic variants.

To accelerate the identification of functional genes underlining QTLs of interest, the development of high-density genetic maps and additional validation through association analysis with marker and trait are attractive approaches. Considering the superiority of QTL and GWAS analyses in the genetic dissection of complex traits, many genes in QTLs associated with plant architecture have been identified in crop plants (Tar’an et al., 2003; Josie et al., 2007; Kumawat et al., 2012; Liu et al., 2013; Stanton-Geddes et al., 2013; Kumar et al., 2014; Mir et al., 2014; Li et al., 2016). Marker-trait association analysis has also been applied recently to horticultural germplasm collections of moderate size. In watermelon, association mapping with iPBS markers identified markers associated with drought-related traits including plant height and stem diameter (Coşkun, 2025), and association mapping with ISSR, SSR, and SRAP markers identified markers associated with salinity-related physiological traits (Coşkun et al., 2025). In this context, the present study used a combinatorial approach to understand the complex genetic architecture of PH traits in pepper, aiming to characterize the loci underlying these traits as a basis for future marker development.

In the present work, we first constructed a genetic map based on 117 F_2_ individuals from the JM population, derived from a cross between the *C. annuum* ‘Jeju’ and ‘Micropep’, to determine the QTLs relevant to PH. Additionally, a genetic map was constructed using 153 F_2_ individuals from the TM population derived from crossing ‘Takanotsume’ and ‘Micropep’ to identify QTLs associated with traits of the main stem. PH was partitioned into MSL, NMSN, and ANLMS because stem elongation and node production are distinct developmental processes. ANLMS was obtained as MSL divided by NMSN, so the three traits separate how long the main stem is from how many nodes it carries and how long each node is. Subsequently, GWAS for the main stem length was performed with the core collection of pepper (Lee et al., 2016) to discover significant trait-associated genes. The core collection was included because a biparental population samples only the two alleles carried by its parents, whereas a diverse panel samples allelic variation across *Capsicum* and allows the linkage results to be compared with association signals from a much wider set of genetic backgrounds. Using high-resolution markers derived from the high-quality *C. annuum* ‘Dempsey’ reference genome (Lee et al., 2022), novel candidate genes responsible for PH and traits of the main stem in pepper were discovered in this study. Our findings provide evidence for a complex inheritance of plant architectural traits and facilitate an understanding of their genetic architecture. The two populations were selected to address complementary phenotypic questions. The marked difference in plant height between ‘Jeju’ and ‘Micropep’ made the JM population suitable for mapping overall PH. The TM population was used to dissect MSL, NMSN, and ANLMS because ‘Takanotsume’ and ‘Micropep’ differed markedly in main-stem architecture despite sharing the same genotype at the *Fasciculate* locus (Elitzur et al., 2009). Because ‘Micropep’ was a parent in both crosses, it provided a common reference for comparing the two populations.

## Materials and methods

### Plant materials and genomic DNA extraction

A pepper core collection (Lee et al., 2016) was used as the association panel. Genotype data were available for 353 accessions, comprising 226 *C. annuum*, 50 C. baccatum, 49 C. chinense, 24 C. frutescens, two C. chacoense, one C. eximium, and one C. praetermissum.

A 117 F_2_ segregation population, ‘JM’ segregation population, was developed from a cross between the *C. annuum* ‘Jeju’ and ‘Micropep’ for QTL mapping of PH traits. The maternal parent ‘Jeju’ is a Korean landrace with a taller height, whereas the paternal parent ‘Micropep’ has a shorter height. Another segregation population, an F_2_ population, ‘TM’ segregation population, derived from a cross between *C. annuum* ‘Takanotsume’ and ‘Micropep’, was used for the QTL analysis of main stem related traits.

Seeds were sterilized using 10% trisodium phosphate and 2% sodium hypochlorite solutions, before sowing in a seed tray with commercial potting mixture (Hanarum, Republic of Korea). GWAS association population accessions were grown in 20 cm wide plastic pots containing commercial potting mixture (Hanarum) in greenhouse conditions at Seoul National University (Suwon, Republic of Korea). Plants were grown under natural photoperiod without supplemental lighting, and no artificial temperature control other than routine greenhouse ventilation was applied. Irrigation and fertilization followed standard greenhouse practice for pepper. Pots were arranged in rows on greenhouse benches, and the same protocol was used for all populations. A total of 117 F_2_ plants of JM population and their parental lines were raised under the same greenhouse conditions in the year 2016. A total of 154 individuals of the F_2_ population derived from ‘Takanotsume’ and ‘Micropep’, with three plants per parent, were evaluated in 2023.

Young leaves of the plants consisting of both segregation and association populations were collected at the seedling stage for genomic DNA (gDNA) extraction using the cetyltrimethylammonium bromide (CTAB) method (Yang et al., 2012). Electrophoresis with 1% agarose gel and a NanoDrop spectrophotometer were used to check the quality and concentration of extracted gDNA, respectively, before restriction enzyme digestion and genotyping-by-sequencing (GBS) library construction.

### Phenotypic evaluation

PH was evaluated for the parental lines, for 117 F_2_ population from the cross of *C. annuum* ‘Jeju’ and ‘Micropep’, and 351 core collection accessions. PH was measured in centimeters (cm) from the soil to the head of the plant at complete maturity. Complete maturity was defined as the stage at which a plant had shifted to reproductive growth and carried set fruit, and at which the main stem had stopped elongation. The main stem, a major component of PH, was subdivided into the following aspects: main stem length (MSL), number of main stem nodes (NMSN), and average node length of the main stem (ANLMS), and phenotyped at complete maturity, in the TM segregation population. MSL was measured from the soil surface to the first branch of the main stem. NMSN was the count of nodes on the main stem up to the first branch; a node was defined as the points where leaves emerge. ANLMS was calculated as MSL divided by NMSN in the TM population and in the core collection. For the core collection, MSL, NMSN, and ANLMS were measured over 3 years from 2015 to 2017. Three plants per accession were scored in each season, and each plant was treated as a separate observation. Accessions with no valid observation for a trait were excluded from the analysis of that trait, which left 351 accessions for PH, MSL, and NMSN and 350 for ANLMS. The representative value used for the association analysis was the BLUP.

### Paraffin sectioning

Internodes were compared between ‘Takanotsume’ and ‘Micropep’ because these two parents carry the same Fasciculate genotype but differ markedly in main stem length. The comparison therefore separates stem elongation from the effect of the Fasciculate locus. Internodes from ‘Takanotsume’ and ‘Micropep’ plants were harvested and fixed in FAA solution (10% formaldehyde, 5% glacial acetic acid, and 50% ethanol) for 24 h. The internodes were dehydrated in a series of ethanol solutions (50, 70, 90, and 100%). For infiltration with paraffin, a series of xylene solutions (25, 50, 75, and 100%) were used. The internodes were soaked in each xylene solution for 1 h and then embedded in paraffin. For sections, tissues were cut with a microtome (HM 340 E, MICROM Lab, Germany), fixed on a slide glass, and dried in an oven at 42°C. Dried tissues were stained with 0.01% Toluidine Blue O. Brightfield photomicrographs were taken using a light microscope (Zeiss Axio Scope with Axiocam 506 camera, Carl Zeiss A.G., Germany). Internodes were sampled from the same node position on the main stem of each parent, and sections were cut transversely. Images of the two parents were acquired at the same magnification and are shown at the same scale. Cell dimensions were not measured, so the comparison between the parents is qualitative.

### GBS library preparation and variant calling

Segregation and association populations were genotyped-by-sequencing to discover SNPs and establish genotypes, as described in previous studies (Han et al., 2016, Han et al., 2018; Siddique et al., 2019). Briefly, GBS libraries were prepared from 50 ng/µL of gDNA, which was subjected to restriction by *EcoR*I/*Mse*I, *Pst*I/*Mse*I, and *Ape*KI/*Mse*I for the core collection and *EcoRI/MseI* for the segregation populations (**Supplementary Table 1**). Double digestion was performed with the corresponding restriction enzymes and buffers according to the manufacturer’s instructions. The fragment size distribution of libraries was checked on an Agilent 2100 Bioanalyzer with the DNA 1000 kit (Agilent Technologies, Santa Clara, CA, USA). Primers designed to amplify the restriction fragments were ligated with selected adapters: “GA” for the JM population and “TA” for the core collection and TM segregation population.

Pooled libraries were sequenced using the Illumina HiSeq2000 and HiSeqX platforms at Macrogen (Macrogen, Inc. Seoul, Republic of Korea). To filter the raw sequencing reads for minimum length (> 80 bp) and quality score (> Q20), CLC Genomics Workbench v6.5 (Qiagen, Aarhus, Denmark) was used for the JM population and the core collection, while fastp (Chen et al., 2018) was utilized for the TM population. Raw reads were aligned to *C. annuum* ‘Dempsey’ reference genome (Lee et al., 2022) using the Burrows-Wheeler Aligner (BWA; Li and Durbin, 2009). Variants were called with the Genome Analysis Toolkit (GATK) HaplotypeCaller in GVCF mode, consolidated with GenomicsDBImport, and jointly genotyped with GenotypeGVCFs. GATK version 4.1.7.0 was used for the core collection, and version 4.4.0.0 was used for the JM and TM populations. Biallelic variants were filtered using thresholds of QUAL ≥ 30, MQ ≥ 30, and SOR ≤ 4. Genotypes with DP < 3 were treated as missing, and SNPs with MAF < 0.05 were excluded. The VCF files were imputed using BEAGLE 5.2 (beagle.21Apr21.304.jar) in default options for the core collection.

### Genetic map construction and QTL analysis

Genetic maps show recombination-based distances between markers in centimorgans (cM), whereas physical maps indicate marker positions in base pairs (bp) on the ‘Dempsey’ reference genome. Genetic maps were used for QTL mapping, and physical maps were used to assess map collinearity and compare the positions of QTLs and GWAS signals.

SNPs with a genotype call rate below 0.70 in the JM population and below 0.90 in the TM population were excluded using VCFtools 0.1.16. No additional genotype-quality or heterozygosity filter was applied, and genotypes in the segregation populations were not imputed. Bin maps were constructed by smoothing genotype calls and detecting recombination breakpoints with a sliding-window approach using in-house Python protocols (Han et al., 2016). A window of 18 markers was used for both populations. Genetic maps for each chromosome within each segregation population were constructed using CarthaGene software (de Givry et al., 2005). Variants were grouped into a single genetic group if the genetic distance between them was < 30 cM and the logarithm of the odds (LOD) value, which indicates the likelihood of genetically linked markers, was > 3.

Distances in the genetic map were calculated using the Kosambi mapping function (Kosambi, 1943). Co-linearity of positions between genetic and physical maps was compared and plotted with a dot-plot diagram for individual chromosomes. Composite interval mapping (CIM) was performed using Windows QTL Cartographer 2.5 (Wang et al., 2012) to identify QTLs and estimate their additive effects and the proportion of phenotypic variance explained (*R*^2^). CIM was performed with a 1-cM scan interval, five background parameters, and a 10-cM exclusion window. Trait-specific genome-wide significance thresholds were determined from 1,000 permutations at the 5% significance level. Peaks exceeding the corresponding thresholds were considered significant QTLs. For a secondary exploratory comparison, the highest observed peak on each chromosome was compared with the distribution of genome-wide maximum test statistics obtained from the 1,000 permutations for the same trait. Regions with 0.05 < empirical P ≤ 0.10 were described as borderline linkage regions. The final graphical representation of the bin map and QTLs was created with MapChart 2.32 software (Voorrips, 2002).

### GWAS for architectural traits

Phenotypes were estimated across 2015–2017 using the mixed model “value ~ year + (1 | genotype) + (1 | genotype:year)”. Year was treated as a fixed effect because 2015, 2016, and 2017 represented the specific experimental years under study rather than a random sample of years from a broader population. This specification adjusted explicitly for systematic differences in trait means among the three years. Genotype and genotype-by-year interaction were fitted as random effects to estimate best linear unbiased predictions (BLUPs). The fixed effects for 2015, 2016, and 2017 were weighted equally when the BLUPs were calculated, so that unequal observation numbers among years did not determine the representative value for an accession. ANLMS was calculated as MSL divided by NMSN for observations from the same accession, year, and plant.

Principal component analysis was performed once on the complete genotype panel of 353 accessions using PLINK 1.9. All 25,126 SNPs retained on chromosomes 1–12 were included, and 20 principal components were calculated using the option “–pca 20 header”. From this common principal component analysis (PCA), the PC1-PC3 scores were selected by accession and included as covariates in each trait-specific GWAS. These three PCs were supplied to GAPIT as external fixed covariates, and GAPIT internal PCA was disabled by setting “PCA.total = 0”. After matching genotype, phenotype, and PC data, 351 accessions were included for PH, MSL, and NMSN, and 350 were included for ANLMS. Two accessions had no valid observation for PH, MSL, or NMSN, and one further accession had no valid observation for ANLMS.

GWAS was performed for PH, MSL, NMSN, and ANLMS using Genome Association and Prediction Integrated Tool version 3 (GAPIT3: Wang and Zhang, 2021). Before imputation, SNPs with a genotype call rate of at least 0.90 and a minor allele frequency of at least 0.05 were retained. Missing genotypes were imputed using Beagle version 5.2 (Browning et al., 2018), after which eight markers assigned to Chr99 were excluded. To account for genetic relatedness among accessions, a genomic relationship matrix was calculated from the complete genotype data using the Zhang method implemented in GAPIT3. Before GWAS, three small negative eigenvalues were set to zero. This adjustment made the matrix positive semidefinite (PSD) and provided a mathematically valid and numerically stable covariance structure for the mixed models.

Seven GWAS models were fitted separately. The models were the general linear model (GLM), mixed linear model (MLM), compressed mixed linear model (CMLM), multiple loci mixed model (MLMM), fixed and random model circulating probability unification (FarmCPU), Bayesian-information and linkage-disequilibrium iteratively nested keyway (BLINK), and settlement of MLM under progressively exclusive relationship (SUPER). The Zhang matrix was included in models with a kinship component. Significant markers were identified separately for each model and compared across models.

The threshold set for the significant marker-trait association was based on modified Bonferroni correction (Bonferroni, 1936). The resulting threshold was *P* ≤ 1.98997 × 10^-6^ (0.05/25,126; $-\log_{10}P$ = 5.701). The Benjamini-Hochberg (BH) FDR method was applied separately to each trait and model, and markers with *q* ≤ 0.05 were considered significant according to the FDR criterion. Markers identified by the Bonferroni and FDR methods were reported separately. The distributions of observed and expected *P* values were examined using quantile-quantile (Q-Q) plots, and genomic inflation factors were calculated.

### Candidate gene function annotation

We used the high-resolution and advanced ‘Dempsey’ reference genome (Lee et al., 2022) to identify candidate genes controlling PH, by analyzing closely linked bins or associated SNPs from QTL and GWAS analyses. LD blocks were identified across the genome separately for the core collection of 353 accessions and for the 226 *C. annuum* accessions within the collection using PLINK 1.9 (Chang et al., 2015). SNPs with a minor allele frequency of at least 0.05 were included, and blocks up to 1 Mb were defined using the confidence interval method (Gabriel et al., 2002). Candidate genes were examined within LD blocks containing significant GWAS markers. The physical positions of these regions were compared with those of the QTLs using the ‘Dempsey’ reference genome. QTL positions were first compared with LD blocks identified in the *C. annuum* subset. When no overlap was detected, the comparison was extended to the full core collection. Protein sequences from the ‘Dempsey’ genome within these regions were subjected to a BLAST search against the reviewed protein database ‘Swiss-Prot’ provided by UniProt (Bateman et al., 2023), to verify gene descriptions.

### Statistical analysis

For the core collection, the descriptive statistics were calculated from the raw observations, in which each accession was summarized as the mean of three plants per season across three growing seasons, whereas the BLUPs were used as the phenotype for the association analysis and for the genetic correlation analysis. For the JM and TM segregation populations, each individual phenotype was used in the analyses. Broad-sense heritability (H^2^) was estimated with the formula; $H^{2}=\sigma g^{2}/\sigma p^{2}\times 100$ (Allard, 1960) and the expected genetic advance (GA) was measured using a formula proposed by Lush (1943); GA as % mean = $\sigma g^{2}/\sigma p^{2}\times \sqrt{\sigma p^{2}}\times k$, where, $\sigma g^{2}$ is genetic, $\sigma p^{2}$ is phenotypic variance, and *k* is selection differential (a constant of 2.06 at 5% selection intensity). ANOVA for phenotypic changes according to the accumulation of QTLs was performed using the ‘aov’ function, and t-test was conducted using the ‘t.test’ function from the ‘stats’ package in R (R Core Team, 2025). Box-plots, frequency distribution, Pearson correlations, and co-linearity analysis were constructed/performed using JMP Pro 13 (SAS Institute, Inc., Cary, NC, USA) and ‘ggplot2’ package (Wickham, 2016) in R.

To estimate the genetic correlation among plant height-related traits, the gcta64 in GCTA v1.95.0 was used (Yang et al., 2011). A genomic relationship matrix was constructed using SNP data (MAF ≥ 0.05) of all individuals in the core collection. The estimation of genetic correlation was performed by using the bivariate GREML (Genomic Restricted Maximum Likelihood) model. To consider the population structure, the top four principal components (PC1-PC4) obtained from principal component analysis were included as covariates. For the trait pairs including MSL, the MSL value was log-transformed to minimize the non-normality of distribution and convergence problem.

## Results

### Phenotypic difference in the parental lines of the segregation populations

Two segregation populations with differences in genetic background were used to identify QTLs related to PH in the JM population and associated with the subdivided traits in the TM population. The JM population was generated by crossing *C. annuum* ‘Jeju’ and ‘Micropep’ (**Figures 1A,B**), whereas the TM population was developed by crossing ‘Takanotsume’ and ‘Micropep’ (**Figure 1C**). ‘Jeju’ and ‘Takanotsume’ have long internodes and tall PH. ‘Takanotsume’ and ‘Micropep’ possess the same *Fasciculate* (*FA*) gene for clustering flowers (Elitzur et al., 2009). Despite having the same *FA* genotype, ‘Takanotsume’ and ‘Micropep’ displayed opposite phenotypes at the main stem and branch. ‘Takanotsume’ has an elongated stem and nodes, whereas ‘Micropep’ has a shorter stem and nodes. F_1_ plants from these crosses exhibited intermediate phenotypic characteristics from the parents (**Figures 1A,C**).

**Figure 1:**
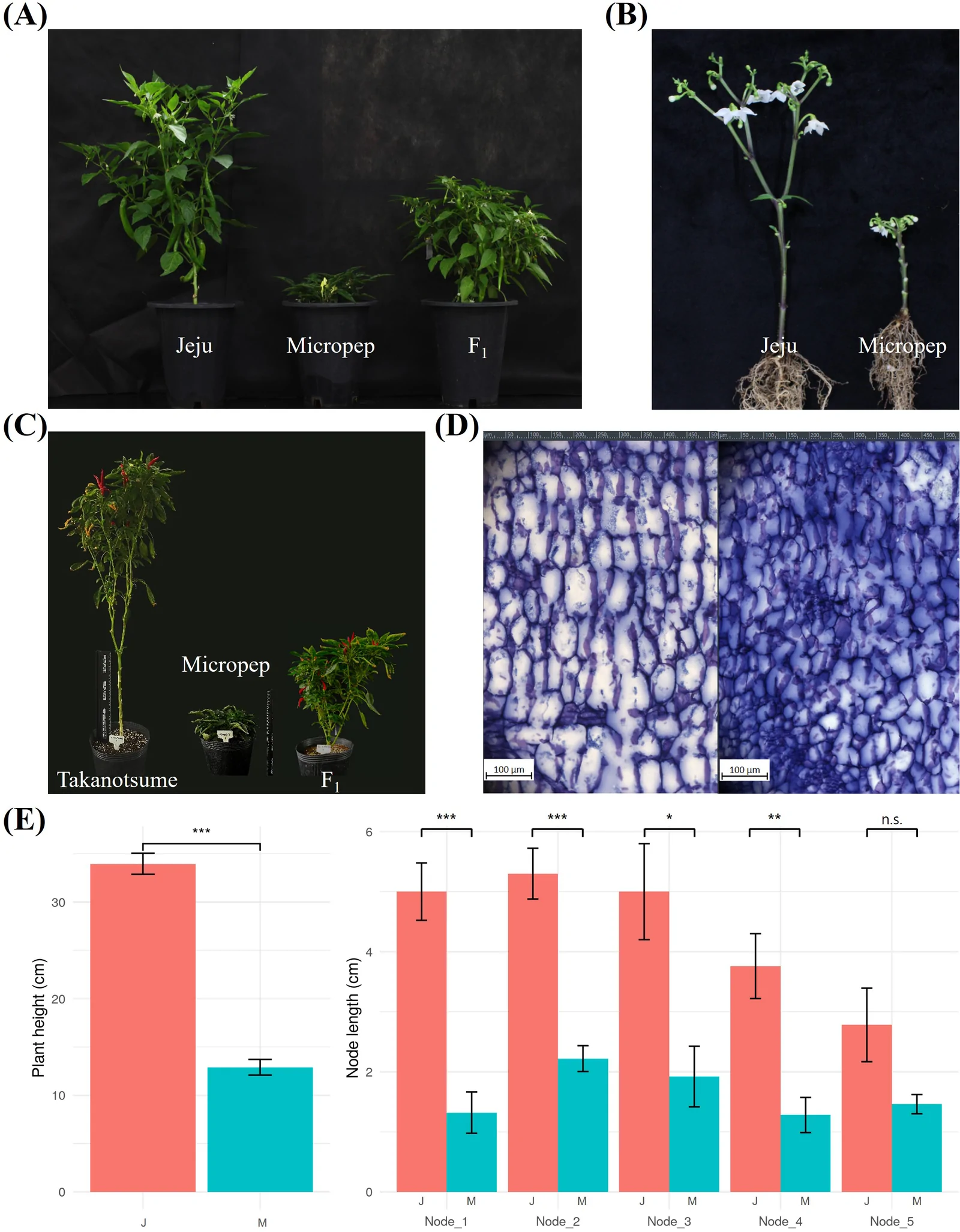
Phenotype of segregation populations used for QTL analysis. The parents exhibited completely distinct phenotypes, with the F_1_ plants demonstrating intermediate phenotypes between the extreme parental phenotypes. **(A)** Parents from the JM panel, ‘Jeju’ and ‘Micropep’, along with their F_1_ offspring. **(B)** Non-clustered and clustered growth habits of parents. **(C)** In the TM panel, parents ‘Takanotsume’ and ‘Micropep’ showed significant differences in plant height, yet both exhibited a clustered growth habit. Their F_1_ progenies also displayed clustered type and had a plant height that was intermediate between ‘Takanotsume’ and ‘Micropep’. **(D)** Brightfield micrographs of transverse internode sections from ‘Takanotsume’ (left) and ‘Micropep’ (right), shown at the same magnification, with a scale bar of 100 μm. Cells in ‘Takanotsume’ were larger and less densely packed compared to those in ‘Micropep’. **(E)** Plant height and internode length of the main stem in ‘Jeju **(J)**’ and ‘Micropep **(M)**’. The x-axis indicated the parent and node information. The y-axis represented the plant height and internode length in centimeters (cm). Different numbers of asterisks were used to denote the degree of significance in length between ‘Jeju’ and ‘Micropep’ (n.s., not significant; *< 0.05; **< 0.01; ***< 0.001).

PH of ‘Jeju’ was 34.18 ± 2.24 cm, whereas that of ‘Micropep’ was 12.95 ± 1.63 cm. Differences in internode length were also observed, and significant differences were found up to the fourth node (‘Jeju’, 3.76 – 5.30 ± 0.42-0.80 cm; ‘Micropep’, 1.28 – 2.22 ± 0.22 – 0.50 cm), but at the fifth node, the node length of ‘Jeju’ significantly decreased, showing no significant difference (‘Jeju’, 2.78 ± 0.61 cm; ‘Micropep’, 1.46 ± 0.16 cm; **Figure 1E**). ‘Takanotsume’ and ‘Micropep’ exhibited MSLs of 29.83 ± 3.01 cm and 6.00 ± 0.87 cm, respectively. For the NMSN trait, ‘Takanotsume’ and ‘Micropep’ displayed 12.67 ± 1.15 nodes and 6.67 ± 1.15 nodes, respectively. ANLMS for ‘Takanotsume’ and ‘Micropep’ were 2.36 ± 0.10 cm and 0.90 ± 0.20 cm, respectively. At the same magnification, internode cells of ‘Takanotsume’ appeared larger and less densely packed than those of ‘Micropep’ (**Figure 1D**). Cell dimensions were not measured, so this comparison is qualitative.

### Descriptive statistics of PH traits and its correlations

Phenotypic variation was evaluated in PH trait in the JM population, in MSL, NMSN, and ANLMS traits in the TM population, and in all traits in the core collection. In the JM population, PH varied from 7.40 to 37.80 cm and exhibited a bimodal distribution, suggesting that PH may be controlled by a few major QTLs and indicating the presence of transgressive segregation (**Table 1**; **Figure 2A**). In the core collection, extensive phenotypic variation in PH was observed, ranging from 14.44 to 221.67 cm, and displayed a normal frequency distribution (**Figure 2B**). The CV for PH was 28.83% with high heritability, indicating a moderate level of variability within this trait. This variability reflects the diverse genetic background of the accessions in the core collection. The skewness for PH was -0.35, suggesting a slightly left-skewed distribution, which may imply a higher frequency of shorter plants. Additionally, the kurtosis was -0.21, indicating a platykurtic distribution, suggesting that the distribution of PH was relatively flat compared to a normal distribution, potentially indicating a more uniform distribution across the observed values (**Table 1**).

**Table 1:** Descriptive statistics and genetic parameters for plant architectural traits in the segregation populations and the core collection.

Population	Size	Trait	Mean ± SD (cm)	Range (cm)	CV (%)	Skewness	Kurtosis	H^2^
JM	117	PH	12.95 ± 1.63 (M) 34.18 ± 2.24 (J) 21.59 ± 7.02 (F_2_)	7.40-37.80	32.52	0.13	-0.89	
TM	154	MSL	29.83 ± 3.01 (T) 6.00 ± 0.87 (M) 15.88 ± 5.98 (F_2_)	1.5-41.5	37.70	1.54	3.11	
		NMSN	12.67 ± 1.15 (T) 6.67 ± 1.15 (M) 13.29 ± 3.48 (F_2_)	2-24	26.17	0.62	1.40	
		ANLMS	2.36 ± 0.10 (T) 0.90 ± 0.20 (M) 1.20 ± 0.32 (F_2_)	0.6-3.0	27.02	1.81	6.29	
Core collection	351	PH	137.81 ± 39.74	14.44-221.67	28.83	-0.35	-0.21	0.91
	351	MSL	24.12 ± 9.56	5.33-67.78	39.62	1.17	2.56	0.94
	351	NMSN	11.49 ± 2.68	4.71-25.33	23.29	0.42	1.29	0.61
	351	ANLMS	2.12 ± 0.69	0.46-4.92	32.54	0.56	1.18	0.59

M, ‘Micropep’; J, ‘Jeju’; T, ‘Takanotsume’; SD, Standard deviation; CV, Coefficient of variation; H^2^, Broad-sense heritability. Descriptive statistics for the core collection were calculated from the raw observations, in which each accession was summarized as the mean of three plants per season across three growing seasons. The BLUPs used for the association analysis were estimated separately from a mixed model and are not shown here. ANLMS for each core collection accession was calculated by dividing its mean MSL by its mean NMSN, which differs from the association analysis, where ANLMS was derived from the same plant in the same season.

**Figure 2:**
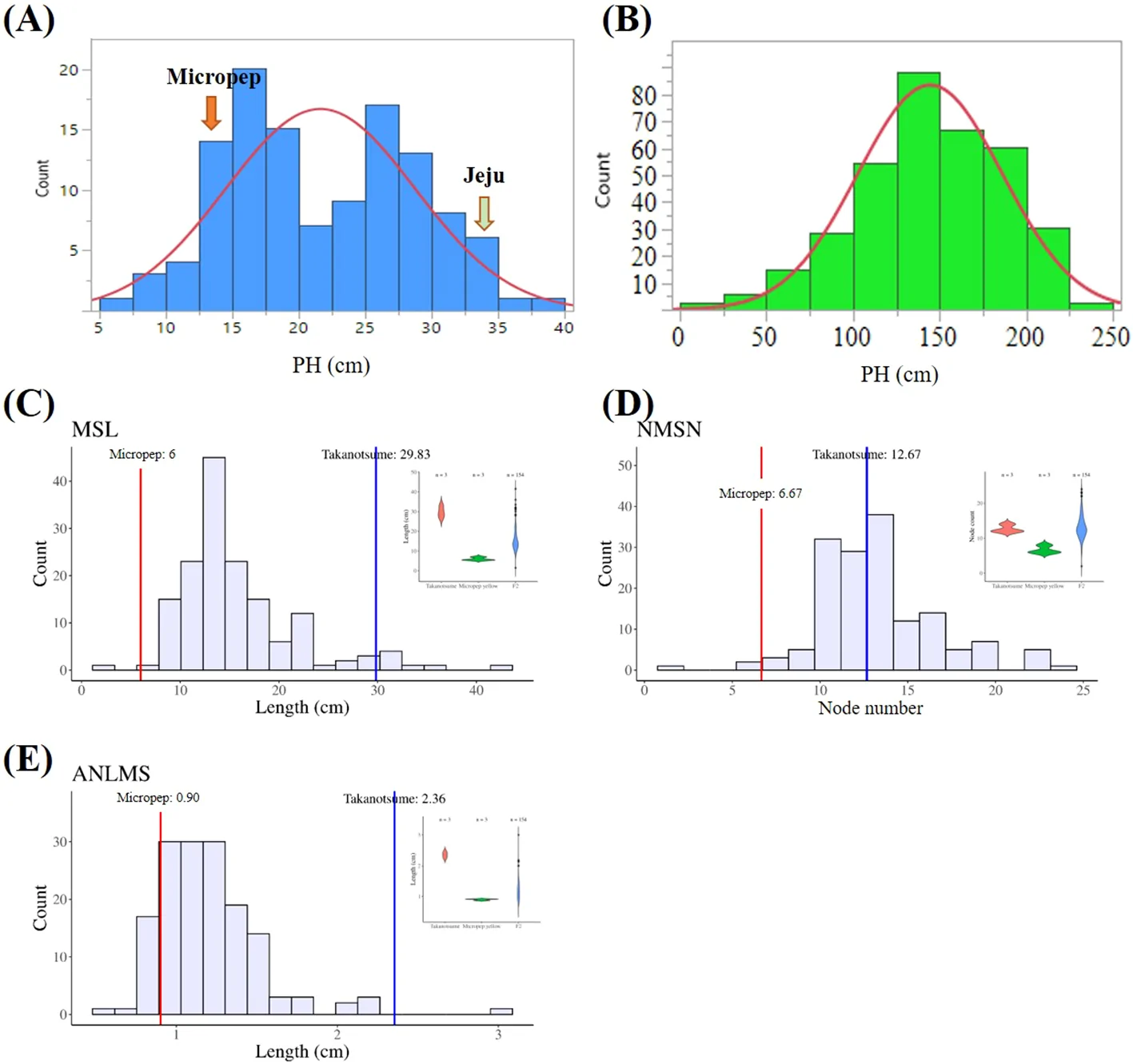
Phenotypic distribution of the segregation populations with parent and association population. **(A)** Frequency distribution of PH in the JM population. The x-axis represented PH in cm and the y-axis showed the counts of individuals. **(B)** Frequency distribution of PH. The x-axis represented PH and the y-axis represented counts in the association population. **(C–E)** Histograms and violin plots displaying the distribution of architectural traits in the TM **(C)**, main stem length; **(D)**, number of main stem nodes; **(E)**, average node length on main stem. In the histograms, the x-axis represents phenotypic values, and the y-axis represents the count of individuals in each range. The red vertical line indicates the phenotypic value of ‘Micropep’, while the blue vertical line indicates that of ‘Takanotsume’. In the violin plots, semi-transparent dots represent outliers.

The parental lines of the TM population, ‘Takanotsume’ and ‘Micropep’, exhibited markedly distinct phenotypes. The F_2_ plants demonstrated a wide range of MSLs, from 1.5 to 41.5 cm, with a mean of 15.88 ± 5.98 cm (**Table 1**; **Figure 2C**). For NMSN, the F_2_ generation varied between having 2 and 24 nodes, with an average of 13.29 ± 3.48 nodes (**Table 1**; **Figure 2D**). In the F_2_ population, ANLMS ranged from 0.6 to 3.0 cm, with a mean of 1.20 ± 0.32 cm (**Table 1**; **Figure 2E**). In correlation analysis among these three traits, MSL exhibited a high correlation coefficient with both NMSN and ANLMS, whereas no correlation was observed between NMSN and ANLMS. These results are consistent with the finding that the MSL is regulated by both the number of nodes and the length of each node (**Supplementary Figure 1A**). The skewness values for MSL, NMSN, and ANLMS were 1.54, 0.62, and 1.81, respectively. The positive skewness indicates a distribution with a tail extending toward higher values, suggesting that a subset of the F_2_ population exhibited traits significantly exceeding the average. Additionally, the kurtosis values for MSL, NMSN, and ANLMS were 3.11, 1.40, and 6.29, respectively. The kurtosis for MSL and ANLMS, in particular, indicates leptokurtic distributions, which are characterized by a pronounced peak and heavy tails, implying that the data are more outlier-prone or that the trait variability tends to cluster around a central value more than a normal distribution does. These statistics suggest that while the F_2_ progeny generally exhibited intermediate phenotypes compared to their parents, significant variability and extremes in the expression of these traits occurred within the TM population. This variability may be indicative of the influence of underlying genetic factors or environmental interactions (**Table 1**).

In the core collection, the MSL ranged from 5.33 to 67.78 cm, with a relatively high CV of 39.62%. The skewness was 1.17, indicating a significantly right-skewed distribution; kurtosis was 2.56, suggesting a leptokurtic distribution. NMSN ranged from 4.71 to 25.33 nodes, with a CV of 23.29%, skewness of 0.42, and kurtosis of 1.29. ANLMS ranged from 0.46 to 4.92 cm, with a CV of 32.54%, skewness of 0.56, and kurtosis of 1.18. A correlation analysis was conducted between PH and the three traits that comprise PH. MSL had the highest correlation with PH, with a Pearson correlation coefficient of 0.60. In contrast, NMSN exhibited the weakest association with PH. MSL also showed a strong correlation with ANLMS, with a coefficient of 0.77 (**Supplementary Figure 1B**).

When comparing the phenotypes between the core collection and TM population, the CVs for MSL in the TM population and core collection were markedly higher at 37.70% and 39.62%, respectively, compared to those for other traits within both populations. The high CVs suggest a significant genetic diversity within the TM population and the core collection for MSL, implying that this trait was subject to substantial genetic control and exhibited considerable phenotypic plasticity across different genotypes. The variability may also indicate that MSL was more sensitive to environmental fluctuations. However, since H^2^ for MSL was high at 0.94, it appears that the increased CV was driven more by genetic diversity than by environmental influences. The ANLMS trait had a higher CV in the core collection (32.54%) compared to the TM population (27.02%), despite having a higher mean value in the core collection. This observation is consistent with the core collection having been scored in three growing seasons, so that inter-annual environmental variation contributed to the observed spread. The inclusion of multi-year data may capture more environmental variance showing an increased CV. Additionally, the low H^2^ observed for the ANLMS trait may indicate greater influence of environmental effects than genetic factors (**Table 1**). On the other hand, the CV for PH in the JM population was higher than that in the core collection, at 32.52%. This difference is probably due to the smaller population size and greater number of relatively short plants in the JM population, resulting in a lower average PH. However, the core collection showed a more normal distribution, with a larger proportion of taller plants (**Figures 2A, B**).

### Genetic correlations among plant height-related traits

To quantify the genetic correlation among plant height-related traits, a bivariate GREML analysis was performed. To correct the effect of population structure, four principal components were used as covariates.

As a result, high positive genetic correlations were observed among the length-related traits (PH, MSL, and ANLMS) (**Figure 3**). In particular, PH-MSL (genetic correlation, r_G = 0.61), PH-ANLMS (r_G = 0.60), and MSL-ANLMS (r_G = 0.94) showed high correlations, indicating that these traits share common genetic factors related to stem elongation. In contrast, the pairs of NMSN with PH and with ANLMS showed lower correlations (r_G = 0.24 and 0.22), and MSL-NMSN was intermediate (r_G = 0.48). Node number therefore appears to be controlled by partly independent genetic factors (**Supplementary Figure 2**).

**Figure 3:**
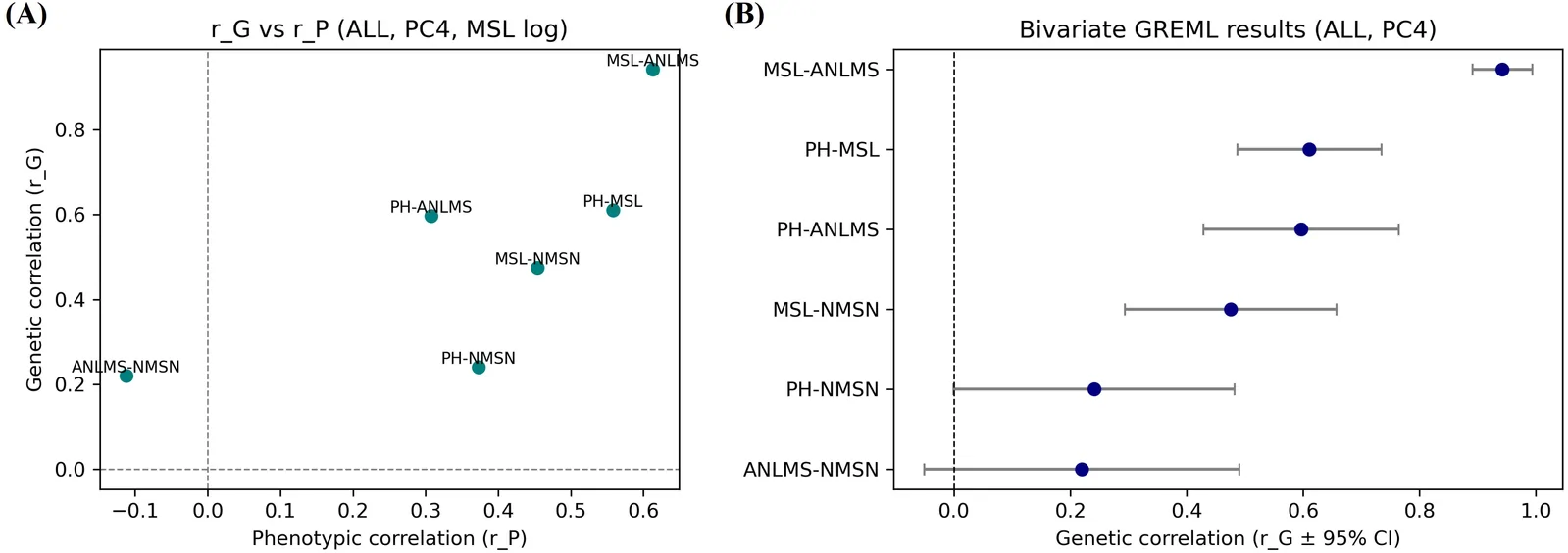
Genetic correlation analysis among plant height-related traits. **(A)** Scatter plot showing the relationship between phenotypic correlation (r_P) and genetic correlation (r_G). The x-axis shows phenotypic correlation of each trait pair, and the y-axis shows genetic correlation. Each point means the correlation coefficient between two traits. When the point is located above the diagonal line (y=x), it means that the genetic correlation of the trait pair is higher than the phenotypic correlation. The top four principal components (PC1-PC4) were included as covariates to correct population structure. For the trait pairs including MSL, log-transformed MSL was used to improve convergence and distribution normality. **(B)** Forest plot showing the estimated genetic correlation and 95% confidence interval (CI) of each trait pair. The y-axis shows the compared trait pairs, and the x-axis shows genetic correlation. The blue dots represent the GREML estimated values, and the gray horizontal lines indicate the 95% CI of each estimate. When the genetic correlation is close to 1, it means a strong positive genetic relationship between two traits. The highest genetic correlations were observed in PH-MSL and MSL-ANLMS pairs, and PH-ANLMS pair also showed significant positive correlation. In contrast, the trait pairs related with NMSN showed relatively low genetic correlations, suggesting that node number might be controlled independently from the stem length related traits.

Genetic correlations were compared with the corresponding phenotypic correlations (**Figure 3A**). The largest differences were found for MSL-ANLMS (r_P = 0.61, r_G = 0.94) and PH-ANLMS (r_P = 0.31, r_G = 0.60), where the genetic correlation was well above the phenotypic correlation. For PH-MSL the two estimates were similar (r_P = 0.56, r_G = 0.61). For PH-NMSN the genetic correlation was lower than the phenotypic correlation (r_P = 0.37, r_G = 0.24). Where r_G exceeded r_P, the difference is consistent with non-genetic variation reducing the phenotypic correlation.

In the forest plot, the 95% confidence intervals (CI) of MSL-ANLMS, PH-MSL, PH-ANLMS, and MSL-NMSN pairs did not include zero (**Figure 3B**). The intervals for PH-NMSN and ANLMS-NMSN included zero, so those two genetic correlations were not distinguishable from zero at this sample size. The heatmap also showed consistent genetic correlations among the three length-related traits. In contrast, the combinations including NMSN showed low correlations, supporting again that node number has a distinct genetic control separated from length-related traits (**Supplementary Figure 2**).

### SNP distribution and co-linearity between genetic and physical maps

A total of 300,263, 1,146,829, and 7,579,731 raw variants distributed across 12 chromosomes, were identified in 117 JM, 154 TM, and 353 association population accessions, respectively, using the *C. annuum* ‘Dempsey’ reference genome (**Supplementary Table 1**; Lee et al., 2022). For QTL mapping, SNPs with genotype call rates of at least 0.70 in JM and 0.90 in TM and a minor allele frequency of at least 0.05 were retained. In the segregation populations, markers that were not polymorphic between the parental lines were additionally removed before bin-map construction. After filtering, 1,066 SNPs in JM and 1,446 SNPs in TM were retained for construction of the bin maps, corresponding to average marker densities of one SNP per 2,784.35 kb and 2,072.11 kb, respectively. The final QTL analyses used 451 bin markers in JM and 518 in TM. The GWAS analysis included 25,126 SNPs on chromosomes 1–12 from 353 accessions, with an average marker density of one SNP per 121.16 kb. In both segregation populations, SNPs were more densely distributed at the terminal regions than at the centromeres, except on chromosomes 6 and 11 in the TM population. The association population showed a higher marker density compared to the segregation populations, because of the use of three restriction enzyme sets (*Pst*I/*Mse*I, *EcoR*I/*Mse*I, and *Ape*KI/*Mse*I) in the construction of the sequence library (**Supplementary Table 1**; **Supplementary Figure 3**).

The total length of the genetic map in the JM population spanned 976.0 cM, whereas that of the TM population spanned 775.9 cM across 12 chromosomes. In the JM population, chromosome 3 was the longest at 133.5 cM, and chromosome 2 was the shortest at 26.3 cM. In the TM population, chromosome 1 was the longest at 113.3 cM, and chromosome 11 was the shortest at 22.4 cM. In the JM population, chromosome 3 had the highest number of SNPs at 121, while chromosome 4 had the fewest at 41. In the TM population, chromosome 11 had the highest number of SNPs at 264, while chromosome 12 had the fewest at 21. The average SNP marker density was 2.8 Mb (2.1 – 6.0 Mb) in the JM population and 2.1 Mb (1.0 – 12.2 Mb) in the TM population. The average bin marker density was 2.2 cM (0.9 – 2.8 cM) in the JM population and 1.5 cM (0.5 – 3.1 cM) in the TM population (**Supplementary Table 2**; **Supplementary Figure 4**).

Comparison of the physical and genetic maps revealed a generally linear relationship across the chromosomes, with an appropriate recombination ratio according to the physical positions. However, some chromosomes displayed an unusual recombination ratio, such as chromosomes 4 and 9 in the JM population, and chromosomes 4 and 8 in the TM population. Additionally, recombination occurred less frequently in the centromere regions; therefore, the genetic position did not increase, even though the physical position increased (**Supplementary Figure 5**).

### QTL analysis for PH traits in segregation populations

Using CIM with LOD score thresholds determined from 1,000 permutations, three QTLs for PH were identified in the JM population, where the genome-wide 5% threshold was LOD 3.80. In the TM population, two significant QTLs were identified, one each for NMSN and MSL. The highest ANLMS peak and two additional MSL peaks had empirical *P* values between 0.05 and 0.10 and were described as borderline linkage regions. In the JM population, three QTLs for PH, named *PH-1.1*, *PH-1.2*, and *PH-11*, were mapped to chromosomes 1 and 11. The major QTL for PH, *PH-11*, which explained 12.86% of phenotypic variation (*R*^2^) with LOD 4.49, was identified at 41.91 cM and spanned a 1.1 Mb region (171.4-172.5 Mb) on the ‘Dempsey’ reference genome. The two minor PH QTL, *PH-1.1* and *PH-1.2*, explained 9.09% and 8.70% of the phenotypic variance with LODs of 5.35 and 7.33, respectively. These were located at 51.71 cM and 63.91 cM, corresponding to 11.7 Mb (190.9-202.6 Mb) and 9.3 Mb (249.6 – 258.9 Mb), respectively (**Table 2**; **Figure 4A**).

**Table 2:** QTLs discovered by composite interval mapping in the segregation populations.

Population	Trait	Chr.	Genetic position (cM)	Physical position (Mb)	LOD	Additive effect	Dominant effect	*R* ^2^
JM	Plant height (PH)	1	51.71	190.9-202.6	5.35	-5.25	-0.71	9.09
		1	63.91	249.6-258.9	7.33	-4.43	-1.63	8.70
		11	41.91	171.4-172.5	4.49	-2.87	1.33	12.86
TM	Number of main stem nodes (NMSN)	3	89.51	276.6-276.8	4.06	1.60	0.27	9.05
	Main stem length (MSL)	1	28.51	76.1-76.6	4.35	2.23	-2.44	16.14

**Figure 4:**
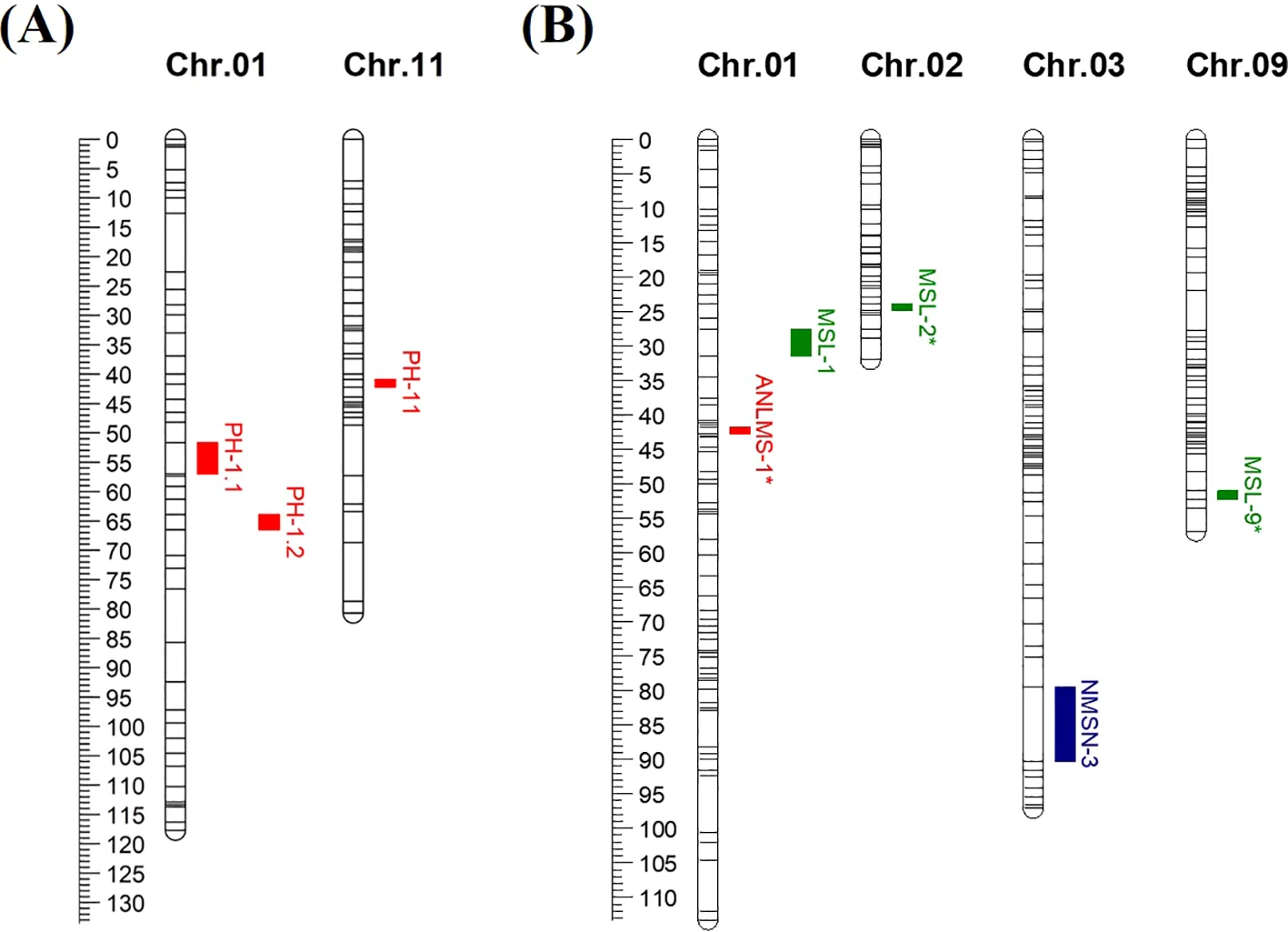
Positions of QTLs for architectural traits on the genetic map. **(A)** QTLs associated with plant height traits on the genetic map of JM population. **(B)** QTLs associated with main stem length (green color), number of main stem nodes (blue color), and average node length of the main stem (red color) traits on the genetic map of TM population. The QTLs are shown on the right-side of the chromosomes. Loci marked with an asterisk are borderline linkage regions (0.05 < *P* ≤ 0.10). The six chromosomes are shown as vertical bars and each horizontal line on the bar represents a single bin marker. The scale on the left side represents genetic distance in cM.

In the JM population, the ‘Jeju’ genotype was designated as the ‘B’ allele when the bin map was constructed. Based on this configuration, we examined how the number of ‘B’ alleles at the three QTLs affected PH. The results indicated that PH tended to increase significantly as the number of ‘B’ alleles increased (*P* = 7.87e-05). However, PH depending on the number of ‘B’ alleles were significantly different only in the groups having four and five ‘B’ alleles, respectively; no significant overall difference was observed. This may be due to the relatively minor phenotypic effect of each QTL individually, making the influence of individual alleles less significant (**Supplementary Figure 6A**).

For NMSN in the TM population, one significant QTL, *NMSN-3*, was detected on chromosome 3. The peak was located at 89.51 cM and had an LOD score of 4.062, exceeding the threshold of 3.476 (empirical *P* = 0.014). The markers flanking the peak were located at 276.6 and 276.8 Mb in the ‘Dempsey’ reference genome. This QTL explained 9.05% of the phenotypic variation and had additive and dominant effects of 1.60 and 0.27, respectively. The positive additive effect suggested that the ‘Takanotsume’ allele increased the number of main stem nodes. The small positive dominance effect indicated a slight increase in heterozygotes relative to the parental midpoint (**Table 2**; **Figure 4B**).

For ANLMS, the highest peak was detected on chromosome 1 at 41.71 cM. The peak had an LOD score of 3.593, which was slightly below the threshold of 3.623 (empirical *P* = 0.052), and was described as a borderline linkage region. The markers flanking the peak were located at 133.2 and 133.5 Mb in the ‘Dempsey’ reference genome. At this peak, the additive and dominance effect estimates were 0.12 and -0.17, respectively, and *R*^2^ was 17.85% (**Figure 4B**; **Supplementary Table 3**).

For MSL, one significant QTL, *MSL-1*, was detected on chromosome 1. It was located at 28.51 cM and had an LOD score of 4.349, exceeding the threshold of 3.577 (empirical *P* = 0.011). The markers flanking the *MSL-1* were located at 76.1 and 76.6 Mb. This QTL explained 16.14% of the phenotypic variation and had additive and dominance effects of 2.23 and -2.44, respectively. The positive additive effect suggested that the ‘Takanotsume’ allele increased main stem length. The negative dominance effect indicated a lower MSL in heterozygotes relative to the parental midpoint (**Table 2**; **Figure 4B**).

Two borderline linkage regions were also observed for MSL. The chromosome 2 peak was located at 23.91 cM and had an LOD score of 3.512 (empirical *P* = 0.057). The markers flanking this peak were located at 155.2 and 158.9 Mb. The additive and dominance effect estimates were 2.36 and 0.31, respectively, and *R*^2^ was 7.63%. The chromosome 9 peak was located at 51.01 cM and had an LOD score of 3.357 (empirical *P* = 0.076). Its flanking markers were located at 265.6 and 267.1 Mb. The additive and dominance effect estimates were 0.74 and 3.12, respectively, and *R*^2^ was 0.79% (**Figure 4B**; **Supplementary Table 3**).

### GWAS for PH traits

GWAS of PH, MSL, NMSN, and ANLMS was conducted using 25,126 SNPs on chromosomes 1-12. The numbers of markers identified using the Bonferroni and BH-FDR criteria differed among the seven models. Q-Q plots and genomic inflation factors showed substantial inflation in several trait-model combinations. Significant-marker counts were therefore interpreted together with the corresponding *P*-value distributions. Genes within LD blocks containing SNPs significant for the Bonferroni threshold were screened for domain architectures related to plant growth (**Supplementary Figure 7**). Genome-wide LD decayed very differently in the two populations. In the entire core collection, mean *r*^2^ remained above 0.2 at all distances up to 10 Mb. In the *C. annuum* subset, restricted to marker pairs in which both markers had a minor allele count of at least 20, mean *r*^2^ fell below 0.2 at approximately 50 kb and below 0.1 at approximately 500 kb. Candidate intervals were therefore defined by linkage disequilibrium blocks rather than by a fixed physical distance, and blocks called in the *C. annuum* subset were examined first.

In the PH analysis, SNP 7_265177454 in chromosome 7 was located within an LD block containing a *Small Auxin-Up RNA* (*SAUR*)-family gene (**Supplementary Figure 7A**; **Supplementary Table 4**). SAUR proteins have been reported to promote auxin-independent hypocotyl elongation in other species (Spartz et al., 2017).

In the NMSN analysis, SNP 6_231136396 in chromosome 6 was located within an LD block containing a *MYB*-related transcription factor with homology to *KUA1*/*KUODA1*, which represses peroxidase genes and modulates cell expansion through reactive oxygen species homeostasis in *Arabidopsis* (Lu et al., 2014), and a separate aminotransferase-like gene whose branch (*ACS*- or *TAR*-related) could not be resolved. SNP 11_277869058 was located within an LD block containing a *LONELY GUY* (*LOG*)-family gene encoding a cytokinin-activating enzyme. In rice, loss of *LOG* function reduces shoot meristem activity and causes premature termination of panicle branch and floral meristems (Kurakawa et al., 2007; **Supplementary Figure 7B**; **Supplementary Table 4**).

In the ANLMS analysis, SNP 4_238813589 in chromosome 4 was located within an LD block containing a gene with homology to *BRI1 KINASE INHIBITOR 1* (*BKI1*), a negative regulator of brassinosteroid signaling that is displaced from the plasma membrane upon brassinosteroid perception in *Arabidopsis* (Wang and Chory, 2006). SNP 9_2324151 was located within an LD block containing a *Small Auxin-Up RNA* (*SAUR*)-family gene (Spartz et al., 2017) and a *GATA*-type zinc-finger gene whose subfamily could not be resolved (**Supplementary Figure 7C**; **Supplementary Table 4**).

For MSL, SNP 2_171524608 in chromosome 2 was located within an LD block containing an alpha-*expansin* gene (family assignment *EXPANSIN-A15*). Expansins loosen the cell wall during stem elongation in other species, although the specific *EXPANSIN-A15* ortholog in *Arabidopsis* is associated with petal and fruit-valve cell expansion rather than vegetative growth (Bernal-Gallardo et al., 2024). The same block contained two *CUP-SHAPED COTYLEDON 2* (*CUC2*)-related *NAC* transcription factor genes. In *Arabidopsis*, *CUC* genes control axillary meristem formation (Raman et al., 2008; **Figure 5**; **Supplementary Table 4**).

**Figure 5:**
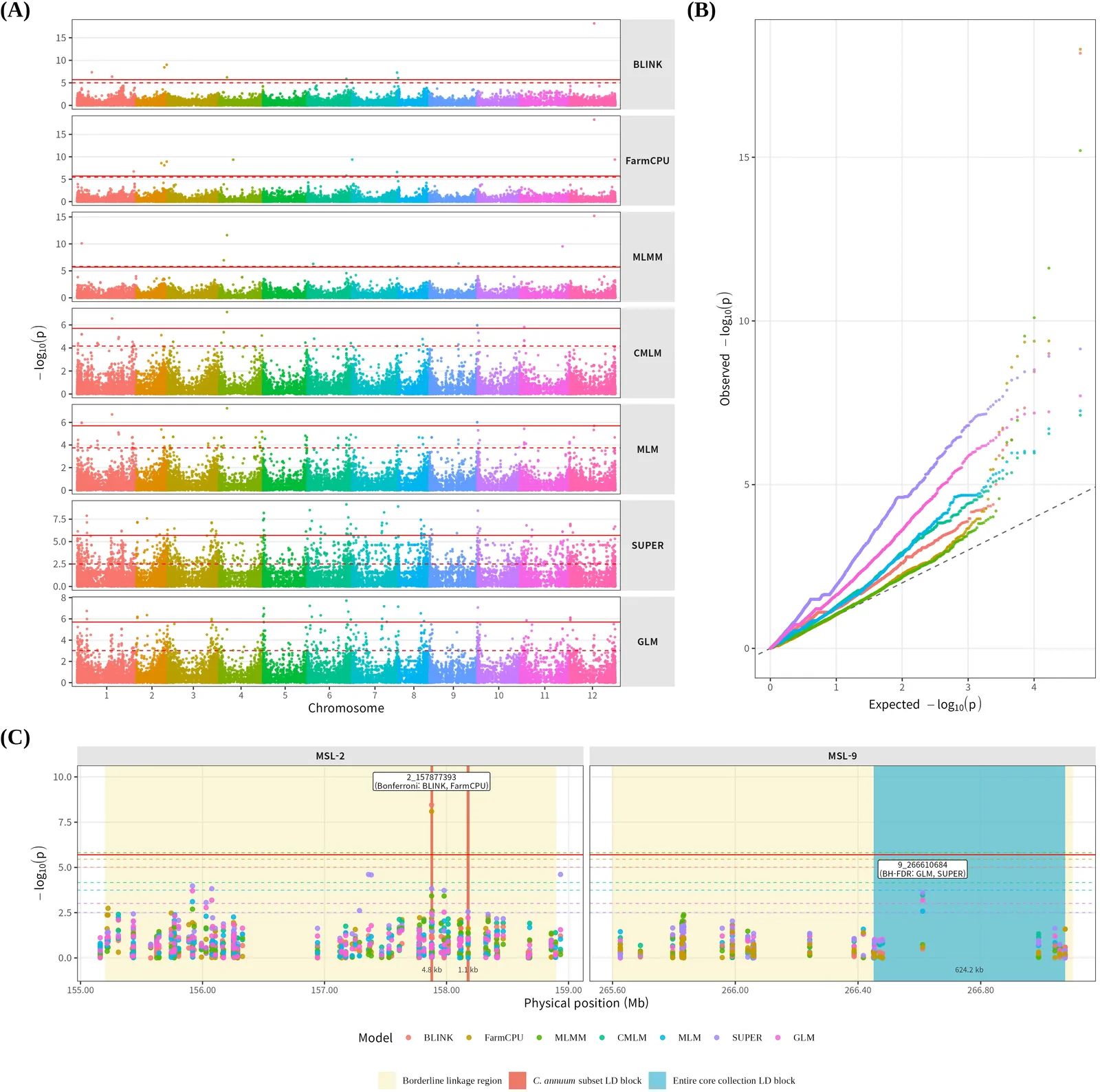
GWAS results for the MSL trait and their positional overlap with a TM-population QTL. **(A)** Manhattan plots for MSL from the seven GWAS models used in this study, shown as separate tracks that share a common chromosome axis. Points are colored by chromosome. The solid red line marks the Bonferroni threshold. The dashed red line marks each model’s own Benjamini-Hochberg FDR threshold. **(B)** Quantile-quantile plot for the same seven models. Colors match the model legend in **(C)**. **(C)** Detailed view of the two loci, MSL-2 on chromosome 2 and MSL-9 on chromosome 9, where a borderline TM-population linkage region overlaps an LD block containing a GWAS-significant SNP for MSL. Points show the same MSL association results as in **(A, B)**, colored by model. The yellow band marks the QTL flanking region. The colored band marks the overlapping LD block, estimated from the *C. annuum* subset (red) or from the entire core collection (blue). The chromosome 2 blocks are far narrower than the flanking-marker interval and are drawn at an enlarged, minimum visible width. Their true size is given by the adjacent kb label.

### Comparison of QTL and GWAS results

GWAS was performed using the entire core collection, whereas LD blocks were estimated separately using genotypes from the *C. annuum* subset and the entire core collection. The significant QTLs *NMSN-3* and *MSL-1* and the borderline linkage regions *ANLMS-1*, *MSL-2*, and *MSL-9* were compared with LD blocks containing significant SNPs from the GWAS. Neither *NMSN-3* nor *MSL-1* overlapped an LD block containing a significant SNP for the corresponding trait in either set of LD blocks. No overlap was found for *ANLMS-1* (**Supplementary Table 3**).

The borderline linkage region *MSL-2* overlapped two LD blocks estimated from the *C. annuum* subset. These blocks extended from 157.877 to 157.882 Mb and from 158.176 to 158.177 Mb, and both contained SNPs that were significant in the GWAS of the entire core collection. In the first block, SNP 2_157877393 was associated with MSL in BLINK, FarmCPU, and SUPER. The BLINK and FarmCPU results met both the Bonferroni and BH-FDR thresholds, whereas the SUPER result met only the BH-FDR threshold. SNPs 2_157877393 and 2_157882226 were associated with ANLMS in SUPER, and SNP 2_157882226 was associated with PH in SUPER after BH-FDR adjustment. In the second block, SNP 2_158176252 was associated with MSL in SUPER, SNP 2_158177322 with ANLMS in SUPER, and SNP 2_158177365 with NMSN in GLM after BH-FDR adjustment. For PH, SNP 2_158176252 was detected by GLM and SUPER, and SNP 2_158177365 was detected by GLM. The SUPER result for SNP 2_158176252 also met the Bonferroni threshold. The first block therefore provided stronger evidence of positional correspondence with *MSL-2*, whereas the associations in the second block and for the other traits were more dependent on the GWAS model (**Figure 4B**; **Supplementary Table 3**).

The borderline linkage region *MSL-9* did not overlap any LD block estimated from the *C. annuum* subset that contained a significant GWAS SNP. However, SNP 9_266610684, which was significant for MSL after BH-FDR adjustment in GLM and SUPER in the GWAS of the entire core collection, was located within this region. In the LD blocks estimated from the entire core collection, SNP 9_266610684 was located within a block extending from 266.451 to 267.075 Mb. The same SNP was also associated with ANLMS in SUPER after BH-FDR adjustment (**Figure 4B**; **Supplementary Table 3**).

Within the LD blocks compared above, the only gene directly overlapped by a significant SNP, ‘DEMF02G23510’, was located within the LD block at 158.176-158.177 Mb, the second of the two blocks overlapping *MSL-2*. This gene was annotated as RNA polymerase II subunit RPB1, an essential component of the core transcription machinery, and was not treated as a candidate for any specific trait. No gene was directly overlapped by the other block overlapping *MSL-2*, at 157.877-157.882 Mb. The LD block overlapping *MSL-9* (266.451-267.075 Mb) contained 20 annotated genes, of which two, ‘DEMF09G28060’ and ‘DEMF09G28070’, were predicted, based on sequence similarity in *Arabidopsis* and rice, to encode cytokinin dehydrogenase/oxidase (CKX, InterPro IPR015345). In rice and *Arabidopsis*, *CKX*-family genes such as *OsCKX2*/*Gn1a* have been linked to reproductive meristem activity and floral or ovule number rather than vegetative internode length (Werner et al., 2003; Ashikari et al., 2005; Bartrina et al., 2011). None of the LD blocks compared in this analysis contained a gene jointly supported by the QTL and GWAS results. The following paragraph reports genes supported by the GWAS results alone.

LD blocks containing Bonferroni- or BH-significant GWAS SNPs for NMSN, ANLMS, PH, or MSL were additionally searched for genes with growth-related domain annotations, using InterProScan v5.78-109.0 (Jones et al., 2014). One such block was on chromosome 2 at 171.5-172.1 Mb, approximately 12.6 Mb from *MSL-2*, and contained ‘DEMF02G31890’, predicted as an alpha-expansin related to EXPANSIN-A15. Expansin-family genes have been associated with internode elongation in rice and poplar (Cho and Kende, 1997), although the characterized function of *Arabidopsis* EXPANSIN15 itself is restricted to flower and fruit development (Bernal-Gallardo et al., 2024). The same block contained ‘DEMF02G31830’ and ‘DEMF02G32060’, predicted as CUP-SHAPED COTYLEDON 2 (CUC2)-related NAC transcription factors. In *Arabidopsis*, this clade initiates axillary meristems and establishes organ boundaries, processes that could plausibly affect node patterning (Raman et al., 2008). A block on chromosome 4 at 237.9-238.8 Mb contained ‘DEMF04G25110’, predicted as a BRI1 KINASE INHIBITOR 1 (BKI1)/MAKR-family protein, a negative regulator of brassinosteroid signaling in *Arabidopsis* (Wang and Chory, 2006). Brassinosteroid signaling promotes cell elongation and could thereby influence internode length. A block on chromosome 6 at 230.5-231.5 Mb contained ‘DEMF06G22540’, predicted as a plant MYB-related, SHAQKYF-class transcription factor. Within the same protein family, this gene was also predicted as a homolog of the *Arabidopsis* cell-expansion regulator KUA1/KUODA1 (Lu et al., 2014). A block on chromosome 7 at 264.9-265.2 Mb contained ‘DEMF07G41370’, and a block on chromosome 9 at 2.3-3.3 Mb, approximately 262 Mb from *MSL-9*, contained ‘DEMF09G00450’. Both genes were predicted as Small Auxin-Up RNA (SAUR) proteins, a family for which constitutive expression increased hypocotyl elongation in tomato (Spartz et al., 2017). A block on chromosome 11 at 277.8-278.8 Mb contained ‘DEMF11G26810’, predicted as a *LONELY GUY* (*LOG*)-family cytokinin-activating enzyme (Kurakawa et al., 2007). Each of these six blocks contained additional genes with domain annotations plausibly related to growth (**Supplementary Table 4**). All predictions above were based on sequence similarity to genes characterized in other species.

## Discussion

This study employed three distinct populations to identify genetic factors associated with four PH traits in pepper. Correlation analysis revealed a high correlation between PH and MSL, and MSL showed a high correlation with ANLMS, indicating that ANLMS is the fundamental trait underlying PH. By employing genome-wide, high-quality SNPs obtained through GBS, we were able to find QTLs linked to these traits. Previous studies have validated the effectiveness of using GBS for trait-linked QTL identification (Han et al., 2016; Siddique et al., 2019). Three PH QTLs were identified in the JM population. In the TM population, QTL analysis identified significant QTLs for NMSN on chromosome 3 and MSL on chromosome 1. Neither QTL overlapped an LD block containing significant GWAS SNPs for the corresponding trait. The exploratory analysis identified borderline MSL linkage regions on chromosomes 2 and 9 that corresponded in position with association signals.

### Relationship between PH and its sub-traits

The correlation analysis between PH and its sub-traits demonstrated strong associations, consistent with previous research. Among the traits, correlation between MSL and PH was the highest, while the correlation between ANLMS and PH was relatively lower. However, the strong correlation between MSL and ANLMS suggests that ANLMS is a key component of MSL, which can be considered a fundamental trait contributing to PH. Previous studies have indicated a strong relationship between PH, internode length, and internode number in various crop species. Correlation analysis in peppers showed high correlations between PH and both MSL and internode length, while the correlation with lateral branch number was comparatively lower. The strong correlation between MSL and internode length further suggests that internode length plays a significant role in determining MSL. However, in this RIL population, a high correlation was also observed between MSL and lateral branch number, implying that internode length is also one of the primary factors contributing to MSL (Han et al., 2016). In tomato, the *SlRR6* gene has been identified as a key positive regulator of PH. The knockout of the *SlRR6* gene resulted in a notable decrease in stem length compared to wild-type plants, predominantly due to the shorter length of the internodes. Conversely, *SlRR6* overexpression led to an increase in PH, accompanied by a greater number of internodes. Furthermore, *SlRR6* overexpression resulted in elevated levels of gibberellic acid (GA) and decreased levels of indoleacetic acid (IAA). Treatment with paclobutrazol (PAC) or IAA restored the increased PH phenotype observed in the *SlRR6*-overexpressed lines (Liu et al., 2023). In soybean (*Glycine max*), overexpression of the *GmTOE4a* gene resulted in a reduction in PH, while the number of nodes remained unaltered. Before flowering, there was no noticeable difference in internode length between wild-type and overexpressed plants. However, following flowering, wild-type plants exhibited longer internodes, while the internode length of *GmTOE4a*-overexpressed plants remained consistent. This discrepancy became increasingly evident as the number of nodes increased (Zhao et al., 2015). In maize, overexpression of the *ZmPIN1a* gene, which regulates auxin transport, resulted in enhanced auxin movement to the roots, leading to longer seminal roots and shorter, denser lateral roots. Consequently, internode length decreased, leading to a reduction in PH. Our results, along with these previous studies, collectively indicate that internode length and number directly influence plant height (Li et al., 2018).

### Genetic correlation and its implication for stem length

The high genetic correlations among PH, MSL, and ANLMS indicate that these traits are controlled by common genetic factors or regulatory networks. This agrees with the previous hypothesis that the plant height in *Capsicum* varies with differences in internode and main stem length. By contrast, the low correlation with NMSN suggests that the node number trait is controlled by a separate genetic mechanism, for example, regulation of meristem activity or distribution of growth hormones, which represent different physiological mechanisms. ANLMS is calculated as MSL divided by NMSN, so part of the correlation between MSL and ANLMS is arithmetic and is not by itself evidence of shared genetic control. The comparisons below therefore rely mainly on PH-MSL and PH-ANLMS, in which the two traits were measured independently.

These results from genetic correlation analysis provided important evidence to decide the direction of further GWAS and QTL analyses. Especially, based on the high r_G between PH and MSL, MSL was selected as a representative trait that can most stably reflect the variation of PH. In addition, in the TM population, plant height was partitioned into MSL, ANLMS, and NMSN to investigate the genetic contribution of each component. The analysis of these three traits indicated that different genetic factors contribute to the variation in internode length and node number. This decomposition allowed us to identify detailed genetic factors that could not be captured when plant height was analyzed as an integrated trait.

No single SNP reached the Bonferroni threshold for more than one trait. Within the two chromosome 2 LD blocks that overlap the MSL borderline linkage region, however, SNPs associated with MSL, ANLMS, PH, and NMSN were all detected after BH-FDR adjustment, and the model in which each association was detected differed among traits. This pattern is consistent with associations with several traits in the same LD block, and it does not show that one variant acts on both stem length and plant height. Trait-specific detection also reflects what each trait measures, because PH is the height of the whole plant, MSL is the length of the main stem, and ANLMS is the mean length of a single node. Differences in allele frequency, LD structure, sample size, model calibration, and adjustment for multiple testing further affect which trait reaches significance at a given locus.

The high genetic correlations such as PH-MSL (r_G = 0.61) and MSL-ANLMS (r_G = 0.94) support that MSL is a representative proxy trait which reflects PH variation in a consistent and stable way. Therefore, the GWAS significant SNPs identified from MSL can be interpreted as biologically meaningful genetic factors related to stem growth. The genetic correlation analysis in this study functioned as a key step to clarify the genetic connection among traits and to improve both efficiency and biological interpretation in the subsequent genetic analyses.

### Comparison of the identified QTLs

Composite interval mapping identified significant TM QTLs for NMSN on chromosome 3 and MSL on chromosome 1. Borderline linkage regions were observed for ANLMS on chromosome 1 and MSL on chromosomes 2 and 9, with empirical *P* values between 0.05 and 0.10. These values may reflect moderate effects that were difficult to detect in the TM population, or sampling variation.

The MSL chromosome 2 region showed the clearest positional correspondence with LD blocks containing significant SNPs for MSL in the *C. annuum* subset. The MSL chromosome 9 region showed weaker correspondence with MSL and ANLMS associations in the entire core collection. Differences in allele frequency, LD structure, sample size, model calibration, and adjustment for multiple testing may contribute to the different association patterns among traits.

Usually, on chromosomes 2-8, several QTLs have been reported by using different pepper genetic backgrounds and with different generations of markers (Chaim et al., 2001a; Dwivedi et al., 2015; Han et al., 2016); however, the reported QTLs were inconsistent. This may be due to architectural traits resulting from various traits, and each trait is governed by polygenes which are largely affected by environmental factors (Lv et al., 2018). Therefore, the potential breeding value of these QTLs is limited because they were only effective in specific genetic backgrounds. Consequently, it is necessary to find the more consistent and stable QTLs using the different genetic backgrounds with saturated genetic maps (Han et al., 2016, Han et al., 2018).

### GWAS-derived candidate genes associated with plant height

Along with development of high-throughput SNP genotyping technology in pepper (Kim et al., 2014; Qin et al., 2014; Cheng et al., 2016), GWAS is mostly applied to unravel the genetic basis of pepper traits such as capsaicinoid content (Han et al., 2016; Nimmakayala et al., 2016b), disease resistance (Zhang et al., 2016; Siddique et al., 2019), peduncle length (Nimmakayala et al., 2016a), dirigent (DIR and DIR-like) proteins (Khan et al., 2018), *Hsp70* family genes (Guo et al., 2016), and fruit traits (Nimmakayala et al., 2016b); however, it has never been applied to study pepper PH traits.

The GWAS results identified LD blocks on chromosomes 2, 4, 6, 7, 9, and 11 containing genes with domain architectures plausibly related to growth. Several point to processes that promote cell elongation and cell wall loosening in other species. A *Small Auxin-Up RNA* (*SAUR*)-family gene was identified for both PH and ANLMS. A *BRI1 KINASE INHIBITOR 1* (*BKI1*) homolog, a negative regulator of brassinosteroid signaling, was identified for ANLMS, and an alpha-*expansin* gene was identified for MSL (**Supplementary Table 4**). Auxin and brassinosteroid signaling promote cell elongation, and expansins loosen the cell wall to allow it. This pattern is consistent with two other results reported above. PH, ANLMS, and MSL showed stronger genetic correlations with one another than with NMSN, and light microscopy of internode sections showed that the taller parent (‘Takanotsume’) had larger and less densely packed cells than the shorter parent (‘Micropep’). The candidate genes for these three elongation-related traits therefore point to a shared, hormone- and cell-wall-driven basis for cell elongation.

By contrast, the candidate gene identified for NMSN was a *LONELY GUY* (*LOG*)-family cytokinin-activating enzyme, associated with shoot meristem activity and panicle branching in rice. Because the number of main stem nodes reflects how many times the shoot meristem initiates a new node, this candidate points toward meristem activity rather than cell elongation. This distinction mirrors the genetic correlation results, in which NMSN showed comparatively low correlation with the other three traits, suggesting a partially independent genetic basis for node number. The MSL block also contained two *CUP-SHAPED COTYLEDON 2* (*CUC2*)-related *NAC* transcription factor genes, a family associated with axillary meristem formation in *Arabidopsis* rather than elongation, alongside the *EXPANSIN-A15* candidate noted above. A *MYB*-related transcription factor and a separate aminotransferase-like gene of unresolved subclade were also identified for NMSN (**Supplementary Table 4**).

### Genetic architecture of plant height and sub-traits

This study drew on three genetically distinct resources. These were the JM population (‘Jeju’ and ‘Micropep’) analyzed for PH, the TM population (‘Takanotsume’ and ‘Micropep’) analyzed for MSL, NMSN, and ANLMS, and the core collection analyzed for all four traits. None of the QTL and GWAS analyses converged on a locus supported by both linkage and association evidence for the same trait. The PH loci detected in the JM population were also on different chromosomes from the PH-associated SNPs identified by GWAS in the core collection. The absence of a shared locus does not by itself indicate that the underlying genetic factors do not exist. It is also consistent with a highly polygenic trait, for which detection depends on the population and method used.

The genetic correlation results reported above offer a more direct way to assess what this design contributed. PH was highly correlated with MSL and ANLMS but weakly correlated with NMSN, which points to at least partially separate genetic control of stem elongation and node number. Measuring MSL, NMSN, and ANLMS directly, rather than inferring them from PH alone, was only possible because the TM population provided the corresponding phenotypes. This decomposition let the elongation-related components be examined apart from node patterning, which a single PH score in one population would not have allowed. Whether the absence of a shared locus across resources reflects this biological separation, limited detection power in populations of this size, or both, cannot be resolved with the present data.

This pattern matches the background dependence already reported for pepper plant-height QTLs, based on comparisons across independent studies with different markers and environments (Chaim et al., 2001b; Dwivedi et al., 2015; Han et al., 2016). Here it appeared within a single design instead. The two segregating populations share ‘Micropep’ as one parent but differ in the second parent and in the traits scored. The core collection panel differs from both in population structure. Three resources built around one project still produced largely distinct significant loci for closely related traits, which suggests that this background dependence is not solely an artifact of comparing separate studies.

Taken together, these results do not point to a locus that can be treated as a general marker for plant height in pepper. The contribution of this design lies elsewhere. Examining these traits across three genetically distinct resources distinguished trait pairs likely to share genetic control (PH, MSL, and ANLMS) from one that does not (NMSN), and showed that the QTL and association signals detected here are tied to the resource in which they were found rather than general across the species.

### *Fasciculate* gene and PH

Previous research on the relationship between the *Fasciculate* (*FA*) gene and PH demonstrated that *FA* homozygous mutants reduced the NMSN, resulting in a shorter MSL and, consequently, a reduced PH. In the previous study, the MSLs of the *FA* homozygous mutants and the homozygous wild-type lines were approximately 25 cm and 31 cm, respectively. Furthermore, homozygous mutants exhibited asymmetric development of the two shoots during the third branching (Elitzur et al., 2009). In the JM population in this study, plants that did not exhibit the fasciculate phenotype like ‘Jeju’ had an average PH of 25.93 ± 5.41 cm, while individuals with the fasciculate phenotype like ‘Micropep’ exhibited an average PH of 15.37 ± 3.36 cm, consistent with the results from previous research. The parents of the TM population, ‘Takanotsume’ and ‘Micropep,’ exhibited highly contrasting PH phenotypes despite having the same *FA* homozygous mutant genotype (**Figure 1C**). The growth patterns observed in ‘Takanotsume’ were similar to those previously described for homozygous mutants, with one extremely short and one elongated shoot asymmetrically developing from new branches. The MSL of ‘Takanotsume’ was also comparable to that observed in the previous study, measuring 29.8 cm. In contrast, ‘Micropep’, despite having the same *FA* genotype, did not produce elongated shoots and exhibited a very short MSL of 6 cm, resulting in an extremely low PH (**Table 1**). This finding suggests the existence of an additional major gene which regulates MSL.

Therefore, in the present study, we aimed to identify other major genes that regulate MSL beyond the influence of the *FA* gene. We created a segregation population using ‘Takanotsume’ and ‘Micropep’, which have the same *FA* mutant genotype but exhibit contrasting PHs, and analyzed traits associated with PH. A borderline linkage region for MSL was detected on chromosome 2. This region overlapped LD blocks containing significant SNPs for MSL in the *C. annuum* subset. *CaGA2ox8* was located near a separate chromosome 2 association signal outside the region between the markers flanking the linkage peak. The *CaGA2ox8* region was located in the ‘Takanotsume’ and ‘Micropep’ genome assemblies by BLASTN, extracted, and aligned with Clustal Omega, which showed a 181-bp deletion in ‘Takanotsume’ approximately 600 bp upstream of the start codon (**Supplementary Figure 8**). The deletion was not genotyped in the core collection, so its relationship to MSL cannot be assessed beyond these two parents, and *CaGA2ox8* is reported here as a gene of interest for future work.

Several limitations apply to these results. The two mapping populations were small, at 117 and 153 individuals, and the intervals between the markers flanking the QTL peaks ranged from 0.2 to 11.7 Mb, so no single gene can be assigned from position alone. The association panel contains seven Capsicum species, and residual population structure cannot be excluded even after fitting three principal components and a genomic relationship matrix. The three resources were grown in different years, and none of the loci reported here was validated in an independent pepper population. 

## Data Availability

The datasets presented in this study can be found in online repositories. The names of the repository/repositories and accession number(s) can be found in the article/**Supplementary Material**.
